# Effect of Amniotic Membrane/Collagen-Based Scaffolds on the Chondrogenic Differentiation of Adipose-Derived Stem Cells and Cartilage Repair

**DOI:** 10.3389/fcell.2021.647166

**Published:** 2021-11-25

**Authors:** Le Cao, Yuling Tong, Xiao Wang, Qiang Zhang, Yiying Qi, Chenhe Zhou, Xinning Yu, Yongping Wu, Xudong Miao

**Affiliations:** ^1^ Department of Orthopedic Surgery, The Second Affiliated Hospital, Zhejiang University School of Medicine, Hangzhou, China; ^2^ Orthopedics Research Institute of Zhejiang University, Hangzhou, China; ^3^ Key Laboratory of Motor System Disease Research and Precision Therapy of Zhejiang Province, Hangzhou, China; ^4^ Department of General Practice, The Second Affiliated Hospital, Zhejiang University School of Medicine, Hangzhou, China; ^5^ Shaoxing Shangyu Hospital of Traditional Chinese medicine, Shaoxing, China

**Keywords:** ADSCs, PRP, amniotic membrane, stent, knee joint

## Abstract

**Objectives:** Repairing articular cartilage damage is challenging. Clinically, tissue engineering technology is used to induce stem cell differentiation and proliferation on biological scaffolds to repair defective joints. However, no ideal biological scaffolds have been identified. This study investigated the effects of amniotic membrane/collagen scaffolds on the differentiation of adipose-derived stem cells (ADSCs) and articular cartilage repair.

**Methods:** Adipose tissue of New Zealand rabbits was excised, and ADSCs were isolated and induced for differentiation. An articular cartilage defect model was constructed to identify the effect of amniotic membrane/collagen scaffolds on cartilage repair. Cartilage formation was analyzed by imaging and toluene blue staining. Knee joint recovery in rabbits was examined using hematoxylin and eosin, toluidine, safranine, and immunohistochemistry at 12 weeks post-operation. Gene expression was examined using ELISA, RT-PCR, Western blotting, and immunofluorescence.

**Results:** The adipose tissue was effectively differentiated into ADSCs, which further differentiated into chondrogenic, osteogenic, and lipogenic lineages after 3 weeks’ culture *in vitro*. Compared with platelet-rich plasmon (PRP) scaffolds, the amniotic membrane scaffolds better promoted the growth and differentiation of ADSCs. Additionally, scaffolds containing the PRP and amniotic membrane efficiently enhanced the osteogenic differentiation of ADSCs. The levels of COL1A1, COL2A1, COL10A1, SOX9, and ACAN in ADSCs + amniotic membrane + PRP group were significantly higher than the other groups both *in vitro* and *in vivo*. The Wakitani scores of the ADSC + amniotic membrane + PRP group were lower than that in ADSC + PRP (4.4 ± 0.44**), ADSC + amniotic membrane (2.63 ± 0.38**), and control groups (6.733 ± 0.21) at week 12 post-operation. Osteogenesis in rabbits of the ADSC + amniotic membrane + PRP group was significantly upregulated when compared with other groups. Amniotic membranes significantly promoted the expression of cartilage regeneration-related factors (SOX6, SOX9, RUNX2, NKX3-2, MEF2C, and GATA4). The ADSC + PRP + amniotic membrane group exhibited the highest levels of TGF-β, PDGF, and FGF while exhibiting the lowest level of IL-1β, IL6, and TNF-α in articular cavity.

**Conclusion:** Amniotic membrane/collagen combination-based scaffolds promoted the proliferation and cartilage differentiation of ADSCs, and may provide a new treatment paradigm for patients with cartilage injury.

## Introduction

The bone joints comprise the articular surface, joint capsule, and joint cavity (Juneja et al., 2020). The articular surface is covered with a layer of smooth cartilage, which aids the movement of bones and mitigates mechanical impact during movement (Tatman et al., 2015). The knee joint of the lower limb bears most of the body weight. The poor regenerative ability of cartilage is a major impediment to the recovery of knee joint damage (Bruns et al., 2018). Chondrocytes exhibit poor self-healing ability. Additionally, cartilage tissue lacks blood vessels and nerves (Armiento et al., 2018). Damage at a size of more than 4 mm will lead to scar healing or nonunion, which adversely affects the structure and function of the cartilage (Massari et al., 2019). Currently used clinical therapeutic strategies for joint damage are not effective. The major treatments for patients with joint damage are long-term symptomatic management and surgical replacement of the joint. However, adjuvant treatment is often needed to mitigate immune rejection after joint replacement surgery (Sturm et al., 2020). Recent advances in stem cell therapy and the widespread application of bioengineering technology have enabled the therapeutic application of stem cell technology in joint injury repair. Auto-transplantation of articular cartilage cells or tissues derived from self-stem cells, such as adipose-derived stem cells (ADSCs), can mitigate immune rejection and promote joint injury repair (Embree et al., 2016).

Adipose tissue, which is the most abundant tissue in the human body, accounts for approximately 20% of the body weight in various mammals (Ulbrich et al., 2018). The advantages of ADSC application in bioengineered stem cell therapy include easy availability and isolation, minimal damage to the donor site, a stable phenotype, high adhesion rate to scaffold materials, strong proliferation, and no marked immune rejection (Storti et al., 2019). In contrast, the disadvantages of autologous chondrocytes include limited availability, increased damage, weak proliferation ability, and the loss of chondrocytic phenotype after *in vitro* culture (Nazempour and Van Wie, 2016). The clinical application of embryonic stem cells is associated with ethical implications (Castro-Viñuelas et al., 2018). Meanwhile, the clinical application of bone marrow stem cells is associated with increased trauma and limited acquisition (Hemmati et al., 2017). Compared with these stem cells, ADSCs are desirable cell engineering materials for *in vitro* culture and transplantation. ADSCs are mesenchymal stem cells with self-renewal and multi-directional differentiation potential (Li and Yue, 2019). Mohammadi-Mahdiabadi-Hasani et al. reported that ADSCs expressed CD29, CD44, CD73, CD90, and CD105 but not CD14, CD31, CD45, and CD235a. C90, a surface marker of stem cells, is an adhesion molecule and a type of membrane surface glycoprotein that functions as a receptor ligand. Previous studies have reported that CD90 is involved in various physiological and pathological processes, such as immune responses, inflammatory responses, coagulation, wound healing, and tumor metastasis (Mohammadi-Mahdiabadi-Hasani et al., 2020). Additionally, CD90 is involved in the recognition, activation, signal transduction, proliferation, differentiation, extension, and movement of cells. Furthermore, CD90 is a biomarker for stem cells (Mildmay-White and Khan, 2017).

Scaffolds are important materials for tissue engineering. Stem cells can adhere to scaffolds, which support the proliferation and differentiation of stem cells (Nair and Tang, 2017). The scaffold materials for cartilage tissue engineering mainly include natural scaffold materials (such as amnion), artificial scaffold materials, and composite materials (Kim et al., 2017). Natural scaffold materials exhibit good bio-compatibility but poor mechanical properties and plasticity (Liu et al., 2019). The advantages and disadvantages of artificial scaffolds are in contrast to those of natural scaffolds (Bae et al., 2018). Currently, composite scaffolds synthesized according to specific proportions and methods are increasingly favored by doctors and researchers. Hortensius et al. (2018) added an amniotic membrane-derived matrix into an anisotropic collagen glycosaminoglycan scaffold to form C/AM composite materials for tendon injury repair. The authors reported that expression levels of pro-inflammatory cytokines were downregulated and that the scaffolds exhibited tendon repair potential. Dewey et al. (2020) reported that mineralized collagen scaffolds fabricated with an amniotic membrane matrix promoted the differentiation of ADSCs into craniofacial bone under inflammatory conditions and the repair of craniofacial bone. Collagen scaffolds used in this study were synthesized from type II and type III collagen, while the amniotic membranes were obtained from pregnant New Zealand rabbits.

Amniotic epithelial cells do not express telomerase and consequently cannot be expanded *in vitro*, which excludes the possibility of tumorigenicity of amniotic epithelial cells in the host after cell transplantation (Ding et al., 2017). The placenta is considered a waste after parturition. Additionally, the use of the amnion is not associated with ethical disputes. The amniotic membrane can be used for clinical applications after obtaining the approval of the ethics committee and the consent of donors (Farhadihosseinabadi et al., 2018). Collagen, a commonly used biological scaffolds, is an artificial scaffold used to repair articular cartilage defects. Collagen maintains the cartilage phenotype, does not elicit immune responses, and promotes the secretion of cartilage matrix (Shekhter et al., 2019).

The extracellular matrix of cartilage mainly comprises type II collagen and glycosaminoglycan (GAG) (chondroitin sulfate and keratin sulfate). Thus, type II collagen and GAG are important indices for evaluating the quality of scaffolds (Whitney et al., 2018). SOX9, an important marker of cartilage differentiation, can directly activate the transcription factors of extracellular matrix protein encoding genes (*COL2A1*, *COL1LA2*, *COMP*, and *ACAN*) in mesenchymal stem cells and promote the formation of cartilage matrix (Kachroo and Vinod, 2020). Raftery et al. demonstrated that the activation of SOX5, SOX6, and SOX9 in mesenchymal stem cells effectively induced their differentiation into cartilage without hypertrophy and inhibited endochondral ossification (Raftery et al., 2020). Alberton et al. confirmed the protective effect of aggrecan on cartilage (Alberton et al., 2019). In aggrecan-deficient mice, the extracellular matrix of articular cartilage exhibited a hard phenotype at 6 months old, while severe cartilage erosion was observed at 12 months old. In this study, we investigated the effects of amniotic membrane/collagen scaffolds on the chondrogenic differentiation of ADSCs and articular cartilage repair

## Materials and Methods

### Isolation and Identification of ADSCs

Three-month-old male New Zealand rabbits weighing 2.0–2.5 kg were administered 15% chloral hydrate and immobilized on a fixed plate (de Lima Santos et al., 2019). The rabbits were sprayed with 75% alcohol for disinfection. The subcutaneous adipose tissue of the groin was excised and the visible blood vessels and fascia tissue were removed. Next, the excised subcutaneous adipose tissues were washed with phosphate-buffered saline (PBS) and cut into pieces. The tissue pieces were digested with collagenase in a centrifuge tube. To obtain a cell suspension, the samples were constantly blown with a straw. The digested samples were centrifuged, and the cells were cultured in high-glucose Dulbecco’s modified Eagle’s medium (Hyclone) supplemented with 10% fetal bovine serum (FBS; Hyclone) and antibiotics in a 5% CO_2_ incubator at 37°C. Immunofluorescence analysis was performed to examine the expression of CD90.

ADSCs were induced using an osteogenic induction medium. Alizarin red staining was performed to confirm the induction of osteogenesis. The individual wells of a 24-well plate were coated with 300 μl of 4% gelatin. The gelatin was absorbed and discarded. The sample was dried using a fan in an ultra-clean table and exposed to ultraviolet radiation for 30 min. After culturing for 24 h, the original culture medium was removed and replaced with the osteogenic induction medium (preheated at 37°C). Osteogenic induction medium was replaced once every 3 days. Next, the medium was removed and the ADSCs were washed with PBS. The ADSCs were fixed with 400 μl of 70% ethanol at room temperature for 1 h, washed thrice with deionized water, and incubated with 300 μl of 1% alizarin red dye solution at 37°C for 30 min. The reaction solution was discarded and deionized water was added to terminate the reaction. Osteogenesis was analyzed under a microscope (Song et al., 2016).

ADSCs were induced with chondrogenic induction medium. The chondrogenic differentiation of ADSCs was confirmed using the toluidine blue staining test (Hashemibeni et al., 2018). The preparation of the 24-well plate and the cell inoculation procedure were the same as described above. After 24 h of ADSC culture, the medium was replaced with chondrogenic induction medium and the cells were cultured for 28 days. The medium was removed and the cells were washed twice with PBS. Next, the cells were fixed with 400 μl of 95% ethanol at room temperature for 15 min. The reaction solution was removed and the cells were washed twice with 500 μl PBS, and stained with the diluted toluidine blue dye solution (Solarbio, G3661) for 30 min. The excess dye was removed and the cells were washed thrice with PBS. The differentiated cells were analyzed under a microscope.

ADSCs were induced with adipogenic induction medium, and the adipogenic differentiation of ADSCs was analyzed using Oil Red O staining (Stojanović et al., 2018). The preparation of the 24-well plate and cell inoculation procedures were the same as described above. After 24 h of ADSC culture, the medium was replaced with adipogenic induction medium (preheated at 37°C before use) and the cells were cultured for 21 days. The culture medium was discarded and the cells were washed twice with PBS and fixed with 400 μl of 4% paraformaldehyde at room temperature for 15 min. The fixative was removed and the cells were rinsed with 400 μl of 60% isopropanol. Next, isopropanol was discarded and the cells were stained with oil red O working solution for 10–20 min. The cells were washed twice with PBS and lipid formation was analyzed using a microscope.

The obtained ADSCs were divided into the following four groups according to different biological scaffolds: CK (ADSCs alone), ADSCs + PRP, ADSCs + amniotic membrane, and ADSCs + amniotic membrane + PRP. The cells in these groups were cultured for several days. The cellular biomarkers were examined on days 7, 14, and 21 of culture.

### Establishment of a Rabbit Model of Articular Cartilage Defect

Three-month-old male New Zealand white rabbits weighing 2.0–2.5 kg were anesthetized by intravenous injection of pentobarbital sodium through the ear vein. A medial incision of approximately 3.5 cm was performed at the knee joint under sterile conditions, and each layer of the subcutaneous tissue was separated to expose the joint capsule. A rabbit model of articular cartilage injury was established by cutting the cartilage layer with a sharp instrument. The rabbits were divided into the following six groups: control (culture medium), ADSC, amniotic membrane, ADSC + amniotic membrane, ADSCs + PRP, and ADSC + amniotic membrane + PRP scaffolds. The Wakitani score was determined and other animal experiments were conducted at week 12 post-operation.

### Scanning Electron Microscopy Analysis of Collagen and Amnion

The structure of biomimetic collagen and amniotic membrane scaffolds were examined using a scanning electron microscope. Type II collagen and type III collagen solutions were diluted with 0.2 M acetic acid solution to concentrations of 4.5 and 0.5%, respectively. Next, type II collagen and type III collagen solutions were mixed and centrifuged for 10 min. To remove bubbles, 100 g of collagen solution was placed in the freezer. Collagen was then injected into the mold. The scaffolds was placed in the freezer for 24 h and placed in a ventilation cabinet to remove the residual acetic acid (Trosan et al., 2018).

To isolate the amnion, several pregnant and parturient New Zealand white rabbits were subjected to cesarean section (Kassem et al., 2018). The amniotic membrane was removed under aseptic conditions. After rinsing with physiological saline repeatedly, the amniotic membrane was incubated with 1% Triton X-100 solution for 24 h in a constant temperature water bath oscillator. Next, the membrane was washed with PBS and incubated with 0.25% trypsin (Beyotime Biotechnology Co., Ltd) and 0.02% EDTA for 4 h at 37°C with agitation. The amniotic membrane was then rinsed with PBS, subjected to freeze-drying, cut into 1 cm × 1 cm pieces, packed and sealed, and disinfected with ethylene oxide. An aliquot of the amniotic membrane was subjected to SEM analysis. The animal experiments were approved by the ethical committee and performed according to the national laboratory animal management regulations.

### CCK8 Was Used to Compare the Chondrogenic Effect and Detect the Proliferation of ADSCs

The effect of different proportions of collagen scaffolds and amniotic membranes on the chondrogenic differentiation of ADSCs was examined using the CCK8 assay. The ADSCs were divided into the following five groups: group A, control (ADSCs alone); group B, ADSCs cultured in the presence of type II collagen and type III collagen type at a ratio of 9.5:1; group C, ADSCs cultured in the presence of type II collagen and type III collagen at a ratio of 9:1; group D, ADSCs cultured in the presence of type II collagen and type III collagen in the ratio of 8.5:1; group E, ADSCs cultured in the presence of amniotic membrane. ADSCs in these five groups were cultured in a 96-well plate in a 5% CO_2_ incubator at 37°C for 24 h. Different proportions of type II and type III collagen were first spread in the individual wells of the plate. Next, the amniotic membrane was placed over collagen. Finally, the ADSCs were seeded on the collagen and amniotic membrane scaffolds. The number of cells in different treatment groups was similar to that in the control group (CK group). The absorbance was measured after 24 h of culture. The cells were then incubated with CCK8 solution (20 μl) in a 5% CO_2_ incubator at 37°C for 1–4 h. The absorbance of the mixture at a wavelength of 450 nm was measured using a microplate reader. The cell growth curve was analyzed based on the measured OD value. A suitable proportion of collagen scaffolds was determined to perform a follow-up experiment on collagen scaffolds (Li et al., 2020).

The four groups of ADSCs from the control (ADSCs), ADSCs + PRP, ADSCs + amniotic membrane, and ADSCs + amniotic membrane + PRP groups were inoculated into a 96-well plate using the same procedure as that used in the CCK8 assay. The absorbance of mixture of cells and CCK8 solution was determined. The absorbance values obtained from the above two experiments are represented using column graphs and compared between the groups.

### Chondrogenic Differentiation of the Cells Was Detected Using Light Microscopy

The images of the cells in the control (ADSCs), ADSCs + PRP, ADSCs + amniotic membrane, and ADSCs + amniotic membrane + PRP groups inoculated in 96-well plates were captured using a microscope.

### Enzyme-Linked Immunosorbent Assay

The levels of TGFB, PDGF, and FGF secreted by PRP were detected using ELISA. PRP was prepared using a two-step centrifugation procedure. Whole blood (18 ml) was collected from the rabbit ear vein using a 20 ml syringe containing 2 ml of sodium citrate. The blood sample was mixed, transferred to a 50 ml centrifuge tube, and centrifuged at room temperature (500 g for 10 min). The centrifuged whole blood was divided into the following three layers: the upper plasma layer, the lower red blood cell layer, and the middle white blood cell layer. The plasma and tunica albuginea layer were transferred to another centrifuge tube and centrifuged at 1,000 g for 10 min. The supernatant was removed to 1 mm above the white blood cell layer and the remaining part was used as liquid PRP (approximately 1 ml). Liquid PRP was incubated with one-fourth the volume of a mixture of 10% CaCl_2_ and 1000 U thrombin to activate the formation of gel PRP scaffolds. PRP scaffolds were incubated with 20 ml of PBS at 37°C, pH 7.4. The concentrations of TGFβ, PDGF, and FGF were detected using commercial ELISA kits, following the manufacturer’s instructions (Irshad et al., 2019).

On day 14, the contents of TGF-β, PDGF, and FGF in the culture medium of the control, amnion membrane + PRP, and amnion membrane + PRP groups were measured using ELISA.

New Zealand rabbits were transplanted with ADSCs from the four groups. At week 12, the rabbits were anesthetized and euthanized by injecting air into their ears. The knee joints were removed, and the cartilage tissue was examined. The contents of GAG and collagen were detected using Blyscan GAG and Sircol Collagen kits, respectively. The levels of IL-1 β, IL-6, TNF-α, TGF-β, PDGF, and FGF in the articular cavity were detected using ELISA.

The following kits were used in this study: TGF-β kit (SOLARBIO product No.: SEKRT-0139), PDGF kit (SOLARBIO product No.: SEKRT-0400), FGF kit (Jianglaibio, JL22069), IL-1β kit (Jianglaibio, JL22053), IL-6 kit (Jianglaibio, JL22058), and TNF-α kit (Jianglaibio, JL10268).

### Toluidine Blue Staining

ADSCs were cultured in collagen (optimal ratio of type II collagen and type III collagen) and amniotic membrane scaffolds for 14 days. Toluidine blue staining was performed to examine chondrogenesis of ADSCs. The wells of a 24-well plate were coated with collagen (optimal ration of type II collagen and type III collagen) and amniotic membranes before cell inoculation. Cells (1 × 10^4^°cells/well) were inoculated into each well and cultured in a 5% CO_2_ incubator (Thermo Scientific 8000) at 37°C. The culture medium was replaced once every 2°days. The cells were stained with toluidine blue (Solarbio G3661) after 14°days of culture. Next, the cells were fixed with 400 μl of 95% ethanol at room temperature for 15 min. Ethanol was removed and the cells were washed twice with 500 μl PBS and stained with diluted toluidine blue dye for 30 min. The excess dye was removed and the cells were washed thrice with PBS (2 min/step). The cells were imaged using an optical microscope (XDS-1A) and an inverted microscope (Olympus, IX71) (Hewer and Schmitt, 2020).

ADSCs cultured on different scaffolds were stained to observe the cell morphology. The cells belonging to the four groups were seeded into a 24-well plate. The wells were coated with scaffolds and seeded with cells (1 × 10^4^°cells/well). The cells were incubated in a 5% CO_2_ incubator at 37°C. The culture medium was replaced once every 2°days. Toluidine blue staining (Solarbio G3661) was performed on days 7 and 14. To perform toluidine blue staining, the cells were fixed with 400 μl of 75% paraformaldehyde (PFA) at room temperature for 15 min. The fixative was removed and the cells were washed twice with 500 μl of PBS and stained with toluidine blue staining solution for 30 min. The excess dye was removed and the cells were washed thrice with PBS (2 min/step) and imaged under a microscope.

The knee joints of New Zealand rabbits were excised at week 12 post-operation and washed with PBS. The cartilage was embedded in paraffin. The sections were stained with toluidine blue (Solarbio G2543) at room temperature for 30 min. The samples were rinsed thrice with distilled water and stained with hematoxylin (Shanghai Zhanyun Chemical Co., Ltd.) for 1 min. The sections were then washed with distilled water and the fluid around the tissue was aspirated. Next, the sections were incubated with anhydrous ethanol. The liquid around the tissue was removed. The sections were sealed using neutral gum and imaged using an inverted microscope (Olympus IX71).

### Quantitative Real-Time Polymerase Chain Reaction

The mRNA levels of COL1A1, COL2A1, COL10A1, SOX9, and ACAN in the control (ADSCs), ADSCs + PRP, ADSCs + amniotic membrane, and ADSCs + amniotic membrane + PRP groups were examined using qRT-PCR. The cells were seeded into 24-well culture plates in a 5% CO_2_ incubator for 24 h. The culture medium was replaced once every 2°days. The samples were collected on days 7 and 14. The cells were digested with 0.25% trypsin and subjected to RNA extraction. To isolate RNA, the cells were incubated with TRIzol reagent for 5 min at room temperature. Next, precooled chloroform (200 μl chloroform/1°ml) was added to the tube containing TRIzol and the solution was vigorously agitated for 15°s and incubated at room temperature for 10 min. The samples were centrifuged at 4°C and the upper aqueous phase (500 μl) was transferred to an RNase-free centrifuge tube and incubated with an equal volume (500 μl) of isopropanol. The RNA pellet was washed with 650 μl of 75% (v/v) ethanol in diethyl pyrocarbonate (DEPC)-treated water. The sample was centrifuged for 5 min and the supernatant was discarded. Reverse transcription was performed. The reaction was performed in a 20-µl reaction mixture comprising 4 μl of 5 × reverse transcription buffer, 0.5 μl of reverse transcription primer, 0.5 μl of downstream universal primer, 0.5 μl of dNTPs, 1 μl of Moloney murine leukemia virus reverse transcriptase, 10 μl of DEPC-treated water, and 4 μl of RNA template. The PCR conditions were as follows: 37°C for 1°h and 95°C for 5 min (to inactivate reverse transcriptase). The qRT-PCR analysis was performed in a 30 μl reaction mixture containing 12.5 μl of SYBRGREEN mix, 0.5 μl forward primer, 0.5 μl reverse primer, 14.5 μl double-distilled water, and 2 μl cDNA template. The PCR conditions were as follows: 94°C for 10°min, followed by 40 cycles of 94°C for 20°s, 55°C for 20°s, and 72°C for 20°s. The data analysis was performed using ABI Prism 7500 SDS software. The relative expression levels were calculated using the 2^−ΔΔCT^ method. The expression levels were graphed using GraphPad (Li et al., 2019; Nunes et al., 2019).

The following primers were used for qRT-PCR analysis:

*GAPDH* (Rabbit)-RT-F: 5′-CTT​CGG​CAT​TGT​GGA​GGG​GC-3′

*GAPDH* (Rabbit)-RT-R: 5′-GGA​GGC​AGG​GAT​GAT​GTT​CT-3′

*COL1A1* (Rabbit)-RT-F: 5′-GCA​TTA​GGG​GAC​ACA​ACG-3′

*COL1A1* (Rabbit)-RT-R: 5′-CCA​GCG​GAC​CCA​ATA​GGA-3′

*COL2A1* (Rabbit)-RT-F: 5′-AAG​TCC​CTC​AAC​AAC​CAG-3′

*COL2A1* (Rabbit)-RT-R: 5′-GTC​TCC​CCA​AAC​CAC​ACG-3′

*COL10A1* (Rabbit)-RT-F: 5′-ACT​CCC​ATT​CCA​TTT​GAT-3′

*COL10A1* (Rabbit)-RT-R: 5′-ACA​GGC​GTG​CCA​TTC​TTA-3′

*SOX9* (Rabbit)-RT-F: 5′-TCG​GTG​AAG​AAT​GGG​CAG-3′

*SOX9* (Rabbit)-RT-R: 5′-GGG​TGG​GGT​GGT​GGT​GTC-3′

*ACAN* (Rabbit)-RT-F: 5′-CCA​GGG​GGG​GTC​GTG​TTC​C-3′

*ACAN* (Rabbit)-RT-R: 5′-TGG​GCT​GCT​GTC​CTT​GTC​G-3′

### Western Blotting

The cells were lysed using radioimmunoprecipitation assay (RIPA) buffer containing phenylmethylsulfonyl fluoride (PMSF) on ice for 2 h. The lysates were centrifuged at 4°C and 12,000 rpm for 10 min. The total protein contents in the supernatant were quantified using bicinchoninic acid (BCA) assay. The absorbance at 562 nm of the mixture was determined using a microplate reader (Thermo, MK3). The standard curve was generated with the absorbance values as ordinate and protein concentration (μg/μl) as abscissa. The protein concentrations (μg/μl) in the test samples were calculated from the standard curve. Equal amounts (25 μg) of proteins were mixed with 3 μl dye solution in a 200 μl EP tube. The electrophoresis conditions were as follows: stacking gel resolution conditions, 80 V for 20 min; resolving gel resolution conditions, 120 V for 1 h. The resolved proteins were transferred to a polyvinylidene membrane. Six pieces of filter paper with the same dimension as that of PVDF membrane were equilibrated with transfer membrane buffer for 15 min. For electroblotting, the voltage was maintained at 100 V for 1 h. Next, the membrane with transferred proteins was blocked with 5% skimmed milk powder at room temperature for 1 h, followed by incubation with the diluted primary antibodies at 4°C overnight. After washing with Tris-buffered saline containing Tween-20 (TBST), the membrane was incubated with the corresponding secondary antibody at 37°C for 1 h. The membrane was washed with TBST. Immunoreactive signals were analyzed using an integrated chemiluminescence imager (Chemiscope 5300 pro) (Li et al., 2019).

### Immunofluorescence Analysis

An immunofluorescence assay was performed to examine the biomarkers of ADSCs. ADSCs cultured for 24 h were fixed with 4% paraformaldehyde for 30 min. The cells were permeabilized with 0.1% Triton X-100 for 10 min, washed with PBS, incubated with 3% H_2_O_2_ in PBS for 15 min at room temperature to inactivate endogenous peroxidase, and washed with PBS. Next, the cells were incubated with 5% FBS (Hyclone) at 37°C for 30 min, followed by incubation with the primary antibodies (anti-CD90; ab225) at 4°C overnight. After washing thrice with PBS for 5 min, the sections were incubated with fluorescein isothiocyanate-labeled secondary antibody (goat anti-rabbit IgG; SA00003-2,1:100) at 37°C for 45°C. The samples were washed thrice with PBS for 5 min and incubated with Hoechst at room temperature for 15 min. Finally, the samples were washed with PBS for 5 min and imaged under a fluorescence microscope (XDS-1A, Olympus IX71).

After 7 and 14 days of culture, ADSCs were fixed with 4% paraformaldehyde for 30 min. The cells were rinsed thrice with PBS (5 min/time), permeabilized with 0.1% Triton for 10 min, and incubated with 3% H_2_O_2_ in PBS for 15 min to inactivate endogenous peroxidase. After washing with PBS (5 min/step), the samples were incubated with 5% FBS (Hyclone) at 37°C overnight. Next, the ADSCs were incubated with the following primary antibodies at 4°C overnight: anti-collagen I (ab6308, 1 μg/ml, Abcam), anti-collagen II (ab3092, Abcam), anti-collagen X (ab58632, 1:300, Abcam), and anti-SOX-9 (ab26414, 1 μg/ml, Abcam) at 4°C overnight. After washing thrice with PBS (5 min/step), the cells were incubated with secondary antibody at 37°C for 45 min. The cells were then washed thrice with PBS (5 min/time) and incubated with Hoechst at room temperature for 15 min. Furthermore, the samples were washed with PBS for 5 min and imaged under a fluorescence microscope (XDS-1A, Olympus, IX71) (Lee et al., 2020).

### Hematoxylin and Eosin Staining

The knee cartilages of the six groups of New Zealand rabbits were sectioned at week 12 post-surgery and rinsed with distilled water. The samples were subjected to hematoxylin staining. The staining duration (5 min) was determined according to the tissue and dye conditions. The tissue was washed with distilled water until the development of a bluish-purple color. For differentiation, the sections were incubated with 1% hydrochloric acid ethanol for 2 s until the development of a red color. The tissue was washed with distilled water until it developed a bluish-purple color. The eosin staining time was controlled according to the tissue staining conditions (approximately 2–8 s). After rinsing with distilled water, the sections were dehydrated using anhydrous ethanol. A drop of neutral gum drop was added to the section and the sections were sealed and imaged under an inverted photographic microscope (Olympus IX71) (Fuchinoue et al., 2020).

### Safranin O Staining

The articular cartilage of New Zealand rabbits was excised at week 12 post-operation and dewaxed in water. The sections were stained with fresh Weigert dye solution at room temperature for 3–5 min and rinsed with running water for 1 min. The acidic medium was used to differentiate the slices for 15 s. The sections were rinsed with running water for 10 min, stained with solid green dye at room temperature for 5 min, rinsed with a weak acid for 10–15 s, stained with saffron at room temperature for 5 min, and rinsed with a weak acid for 10–15 s. Next, the sections were dehydrated using anhydrous ethanol, sealed with neutral gum, and imaged under an inverted fluorescence microscope (Olympus IX71) (Alibegović et al., 2020).

### Immunohistochemical Staining

The articular cartilage was excised at week 12 post-operation. The samples were incubated twice with xylene for 20 min and dehydrated in an alcohol series (100% ethanol, 95% ethanol, 90% ethanol, 80% ethanol, and 70% ethanol; 10 min/step). Next, the samples were washed with 2 ml of PBS using a 1 ml pipette and incubated with antigen repair solution in a microwave oven maintained at a low temperature for 20 min. After washing thrice with PBS (5 min/step), the sections were incubated with 3% H_2_O_2_ in PBS for 10 min at room temperature to completely inactivate endogenous peroxidase, followed by washing thrice with PBS (5 min/step). Furthermore, the samples were incubated with 5% bovine serum (BSA) blocking solution at 37°C for 1 h, followed by incubation with the primary antibodies diluted in 5% BSA at 37°C for 2 h. The samples were then washed and incubated with the species-specific secondary antibodies at room temperature for 40 min. Immunoreactive signals were developed by incubating the sections with 3,3′-diaminobenzidine solution (50 μl) for 5 min. The sections were washed with 2 ml of PBS, incubated with one drop of hematoxylin for 1 min, and washed with running water for 1 min. Next, the sections were incubated with anhydrous ethanol. Ethanol was evaporated and the samples were sealed with one drop of neutral gum. The positive expression sites were observed under a microscope (Olympus IX71), and the images were captured at ×200 magnification (Yagi et al., 2020).

### Statistical Analysis

Three to four biological repeats (including Western blot samples, qRT-PCR, and all the biochemistry staining sections) were used for each experiment, and three technique repeats were used for qRT-PCR. Four to six fields were randomly captured for every section. All values are represented as mean ± standard deviation. The differences were considered significant at *p* < 0.05. All statistical analyses were performed using SPSS (version 16.0; SPSS, Inc., IL, United States). GraphPad Prism 8.0 (GraphPad software, La Jolla, CA, United States) and ImageJ software were used for graphing and image data analysis. The means between two groups were analyzed using two-tailed Student’s *t*-test, while those between multiple groups were analyzed using analysis of variance, followed by Tukey’s post-hoc test. Minimal significant differences were detected using multiple intergroup comparisons.

## Results

### Culture and Differentiation of Adipose Tissue *In Vitro*

**FIGURE 1:**
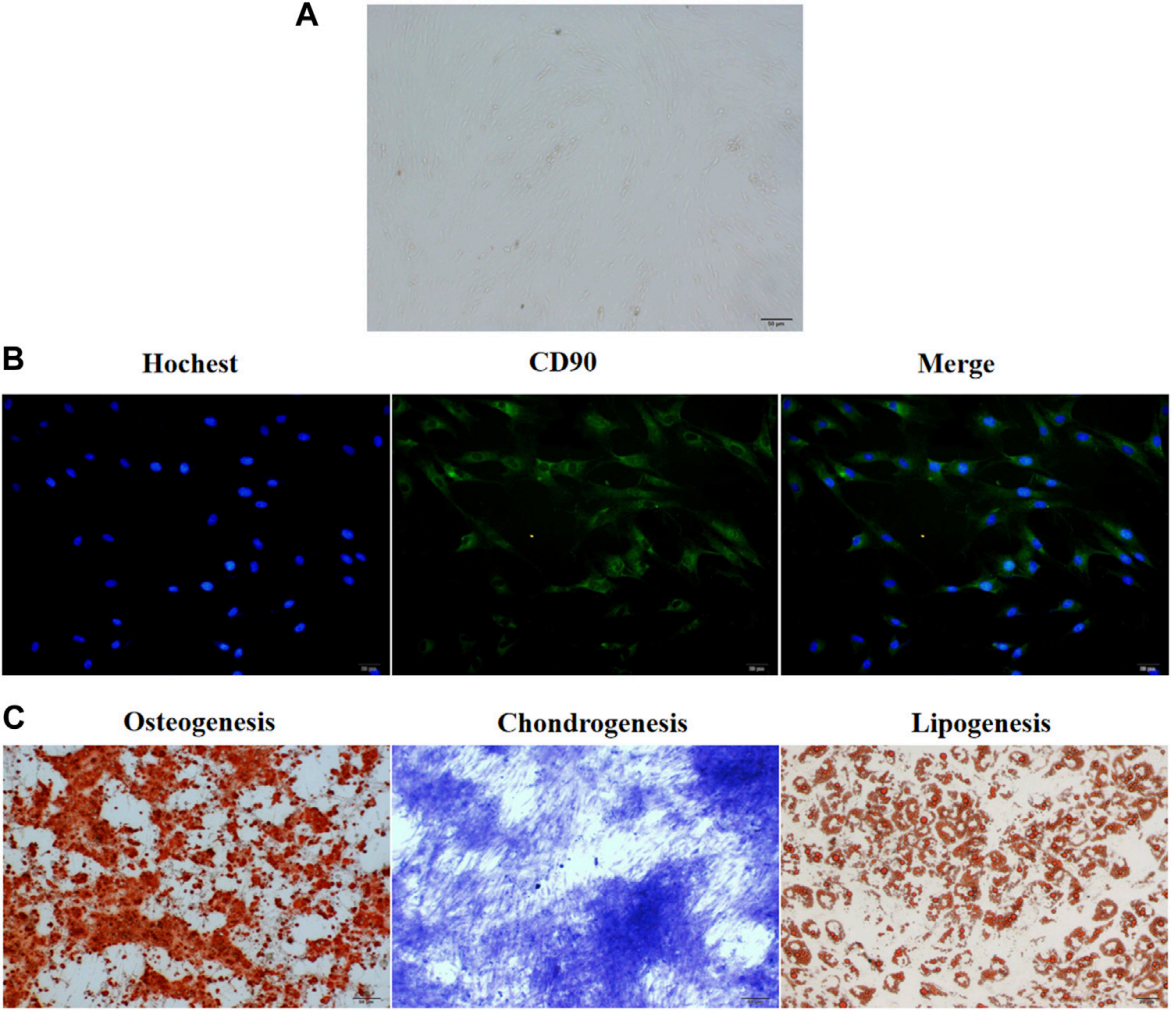
The culture and *in vitro* differentiation of ADSCs isolated from the groin of male New Zealand rabbits. **(A)** ADSCs isolated from rabbits in New Zealand were cultured *in vitro* for 3 days and the cellular morphology was captured by an optical microscope. The representative picture were displayed. **(B)** Immunofluorescence assay showed that CD90 was expressed in more than 90% of ADSCs isolated from New Zealand rabbits. *n* = 3. **(C)** The three-way differentiation ability of ADSCs into osteoblasts, chondrocytes, and adipocytes. Hoechst is a blue fluorescent dye that can penetrate the membrane of living cells and stain the nucleus, *n* = 3.

The groin subcutaneous adipose tissue of New Zealand rabbits exhibited stagnated growth and a small and round-shaped morphology after 24–48 h of culture. After 48 h of culture, the cells expanded and gradually exhibited a short and spindle-shaped morphology. Light microscopy analysis revealed the presence of ADSCs, which exhibited a round and spindle-shaped morphology, after 3 days of culture (Figure 1A). After 5–6 days of culture, the cells formed colonies. The results of the immunofluorescence assay revealed that more than 90% of ADSCs expressed CD90 (Figure 1B). Alizarin red staining revealed a large number of mineral nodules on day 21 post-induction. This indicated the osteogenic differentiation of ADSCs. Toluidine blue staining revealed the differentiation of ADSCs into cartilage on day 28 post-induction. Oil red O staining revealed the presence of a large number of lipid droplets in ADSCs after 21-day post-induction, which indicated the successful induction of adipogenesis in ADSCs (Figure 1C).

### Scaffolds With the Combination of Amniotic Membrane and PRP Effectively Promoted the Growth and Differentiation of ADSCs

**FIGURE 2:**
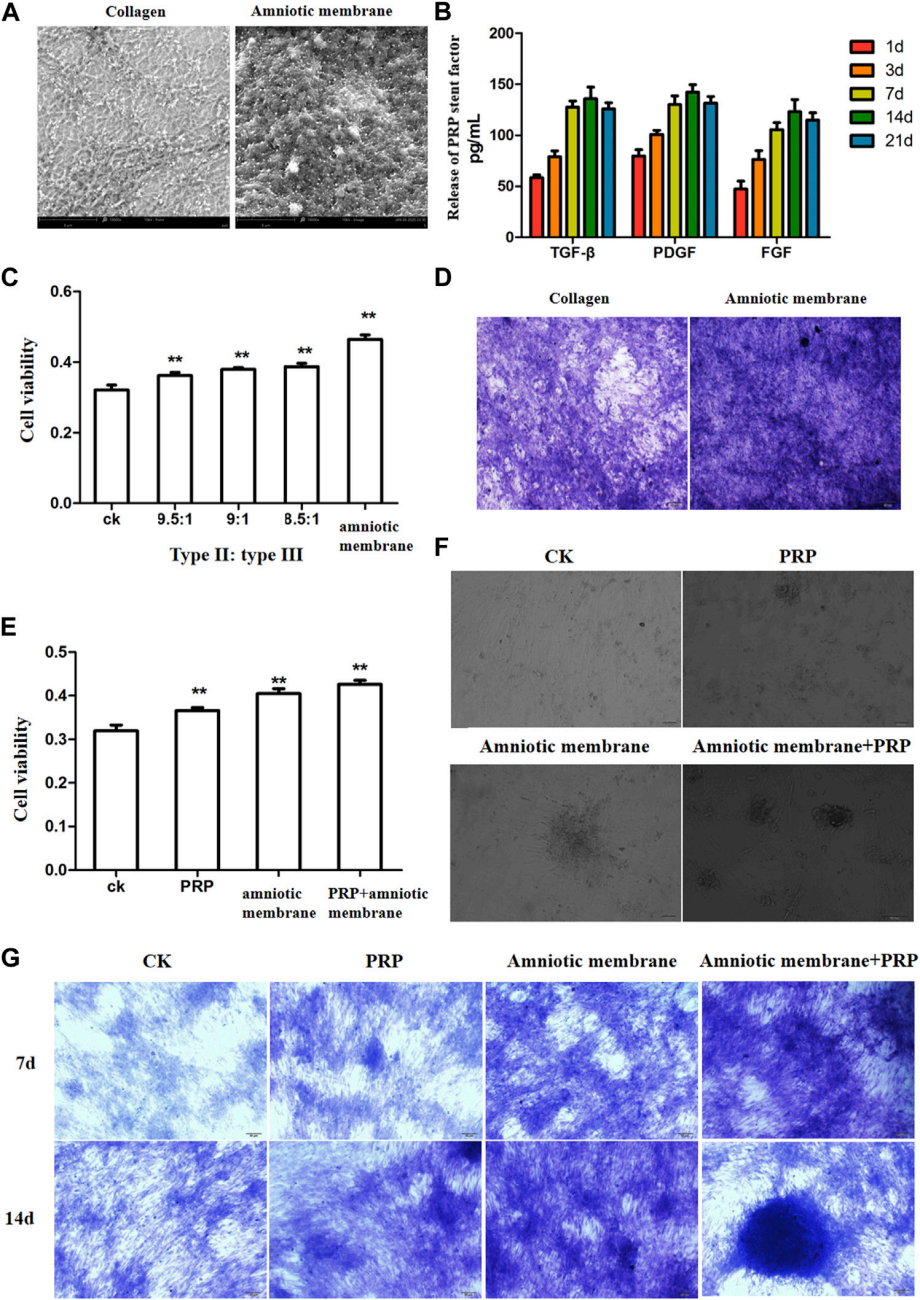
The combination of amniotic membrane and PRP scaffolds showed the best effect for stem cell transplantation, growth, and differentiation. **(A)** Comparison between collagen scaffolds and amniotic membrane scaffolds under a scanning electron microscope. The surface of the amniotic membrane scaffolds is dense, *n* = 3. **(B)** The secretions of various factors in PRP were detected by ELISA at different time points. The results showed that the release of TGF-β, PDGF, and FGF increased with the extension of time, and the increase slowed down at the 14th day, and the decrease trend weakened at the 21st day, *n* = 4. **(C)** CCK8 was used to detect the effect of different proportions of collagen and amniotic membrane scaffolds on ADSC proliferation. The amniotic group had the highest cell activity. **p < 0.01. Control: ADSC. *n* = 3. **(D)** The toluidine blue staining assay showed that the effect of cell cartilage formation in the amniotic membrane scaffolds group was better than that in collagen, *n* = 4. **(E)** CCK8 assay was used to detect the proliferation of ADSCs under different treatments. The ADSC in the PRP + amniotic membrane group showed the fastest proliferation and the best vitality. CK: ADSC alone; PRP: PRP + ADSC; amniotic membrane: amniotic membrane + ADSC; PRP + amniotic membrane: ADSC + PRP + amniotic membrane. **p < 0.01. **(F)** The growth of ADSCs was observed through an optical microscope. The results showed that amniotic membrane + PRP scaffolds well promoted the clustered growth of ADSCs. , *n* = 4. **(G)** The growth and differentiation of ADSCs were detected by toluidine blue staining at different time points. The cells in the amniotic membrane + PRP scaffolds group exhibited the best growth and differentiation. On the 14th day, chondrocytes were induced, *n* = 3.

SEM analysis revealed that the amniotic membrane scaffolds were more compact than the type II/III collagen scaffolds. Additionally, the surface of the amnion membrane exhibited increased concave and convex areas, which provided a good environment for cell adhesion (Figure 2A). The ELISA results revealed that the release of TGF-β, PDGF, and FGF from PRP increased with time. However, the release of these factors slowed down on day 14 and decreased slightly on day 21. These findings indicate that the release of TGF-β, PDGF, and FGF from PRP decreased with time (*p < 0.05) (Figure 2B). The results of the CCK8 assay revealed that the amniotic membrane group exhibited the highest promotion on ADSC proliferation than control (ADSCs alone) and any of collagen II and III combination groups (**p < 0.01) (Figure 2C). Toluidine blue staining revealed that the chondrogenic effect of the amniotic membrane group was higher than that of the collagen group. The ADSCs were densely arranged in the amniotic membrane group (Figure 2D). Therefore, the amniotic membrane was selected to replace collagen as a cell scaffold to induce cartilage formation. The results of the CCK8 assay revealed that the ADSC + amniotic membrane + PRP group exhibited the highest ADSC proliferation and viability rates, followed by the ADSC + amniotic membrane, ADSC + PRP, and control groups (ADSCs alone) (Figure 2E). Light microscopy analysis revealed the presence of chondrogenic clusters in the ADSC + amniotic membrane + PRP, ADSC + amniotic membrane, and ADSC + PRP groups. However, the cartilage was distinctly observed in the amnion + PRP group (Figure 2F). Toluidine blue staining revealed that PRP and amniotic membrane can induce the differentiation of ADSCs into cartilage. On day 14, chondrocytic differentiation was observed in the ADSC + amniotic membrane + PRP group (Figure 2G).

### mRNA Levels of *COL1A1*, *COL2A1*, *COL10A1*, *SOX9*, *ACAN*, and The TGF-β, PDGF, and FGF Levels Were Upregulated in the ADSC + Amniotic Membrane + PRP Group

**FIGURE 3:**
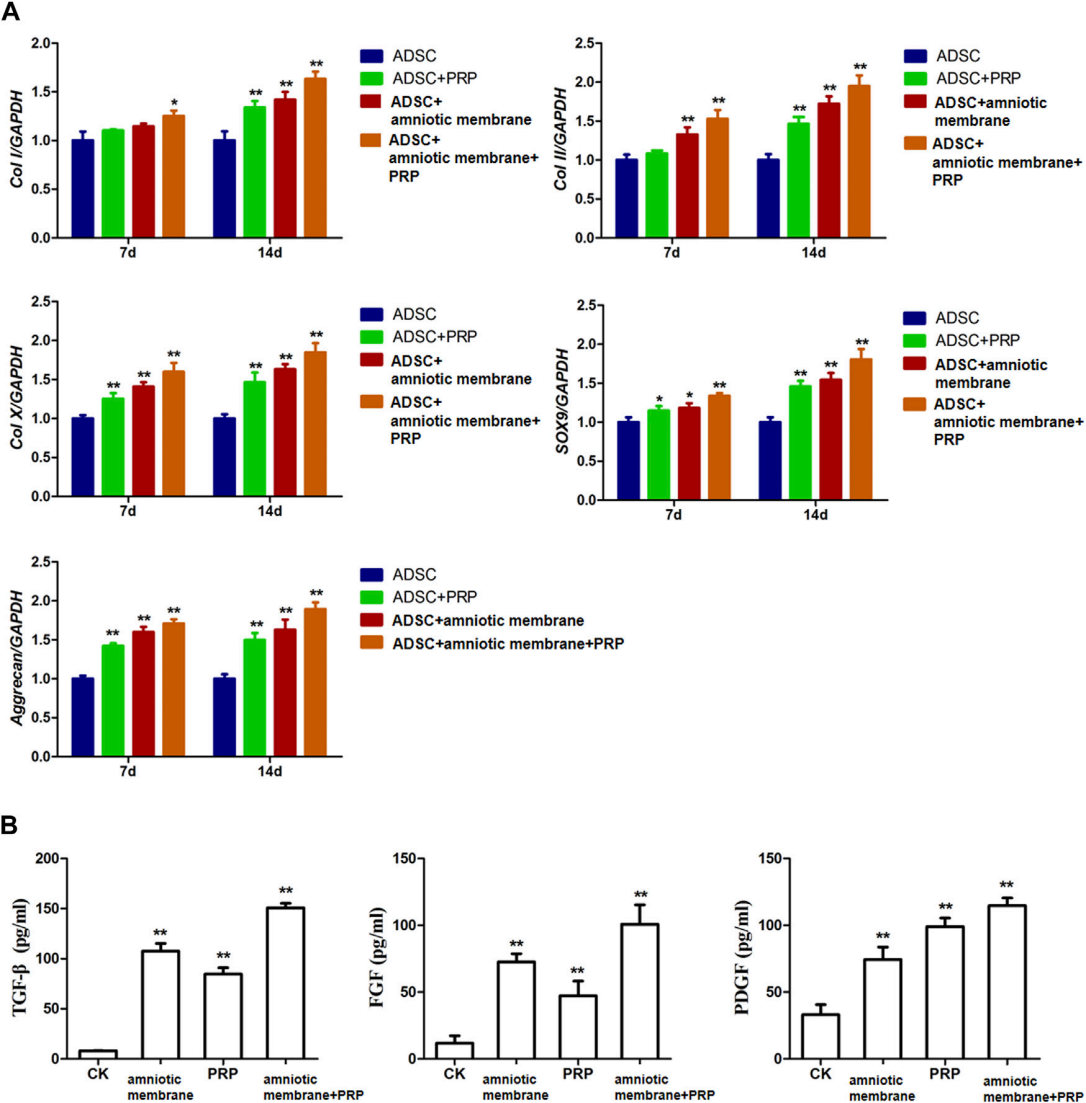
The combination of amniotic membrane and PRP scaffolds exhibited the highest increase of factors related to cell growth and differentiation in ADSCs in transcriptional level. The experiment was divided into four groups: ADSC group, ADSC + PRP group, ADSC + amniotic membrane group, and ADSC + amniotic membrane + PRP group. The expression of various factors in each group was detected at 7 and 14 days after the same culture conditions. **(A)** RT-PCR was used to detect the mRNA expression of collagen I/II/X, SOX-9, and aggrecan. On day 7 and day 14, the mRNA transcription levels of Col I, Col II, Col x, SOX9, and Aggrecan genes were ADSC + amniotic membrane + PRP group > ADSC + amniotic membrane group > ADSC + PRP group > ADSC group. At the 14th day, the transcription levels of these genes in the four groups were significantly higher than those on the 7th day. *p < 0.05, **p < 0.01. Five biological repeats were included in every group. CK: ADSC alone, *n* = 5; PRP: PRP + ADSC, *n* = 5; amniotic membrane: amniotic membrane + ADSC, *n* = 5; PRP + amniotic membrane: ADSC + PRP + amniotic membrane, *n* = 5. **(B)** The expressions of TGF-β, PDGF and growth factor FGF in ADSC cultured on day 14 were detected by ELISA. The expression levels of TGF-β and FGF in ADSC cultured with amniotic membrane + PRP scaffolds were significantly higher than those in other groups. **p < 0.01. CK: ADSCs alone, *n* = 5; PRP: PRP + ADSC, *n* = 5; amniotic membrane: amniotic membrane + ADSC, *n* = 5; PRP + amniotic membrane: ADSC + PRP + amniotic membrane, *n* = 5.

The expression levels of various genes in the four groups were analyzed using qRT-PCR and ELISA. The mRNA levels of *COL1A1*, *COL2A1*, *COL10A1*, *SOX9,* and *ACAN* were significantly upregulated in the ADSC + amniotic membrane + PRP group. Additionally, the expression levels of these genes increased significantly with the duration of culture. The ADSC + amniotic membrane + PRP group exhibited the highest expression levels of these genes, followed by the ADSC + amniotic membrane, ADSC + PRP, and control groups (ADSCs alone) (Figure 3A) The ELISA results revealed that the levels of growth factors were upregulated in the culture medium of the ADSC + amniotic membrane + PRP group. Compared with those in the culture medium of the ADSC + PRP group, the TGFβ and FGF levels were significantly upregulated and the PDGF levels were significantly downregulated in the culture medium of the ADSC + amniotic membrane group (**p < 0.01; Figure 3B).

### Protein Levels of COL1A1, COL2A1, COL10A1, SOX9, and ACAN Were Significantly Upregulated in the ADSC + Amniotic Membrane + PRP Group

**FIGURE 4:**
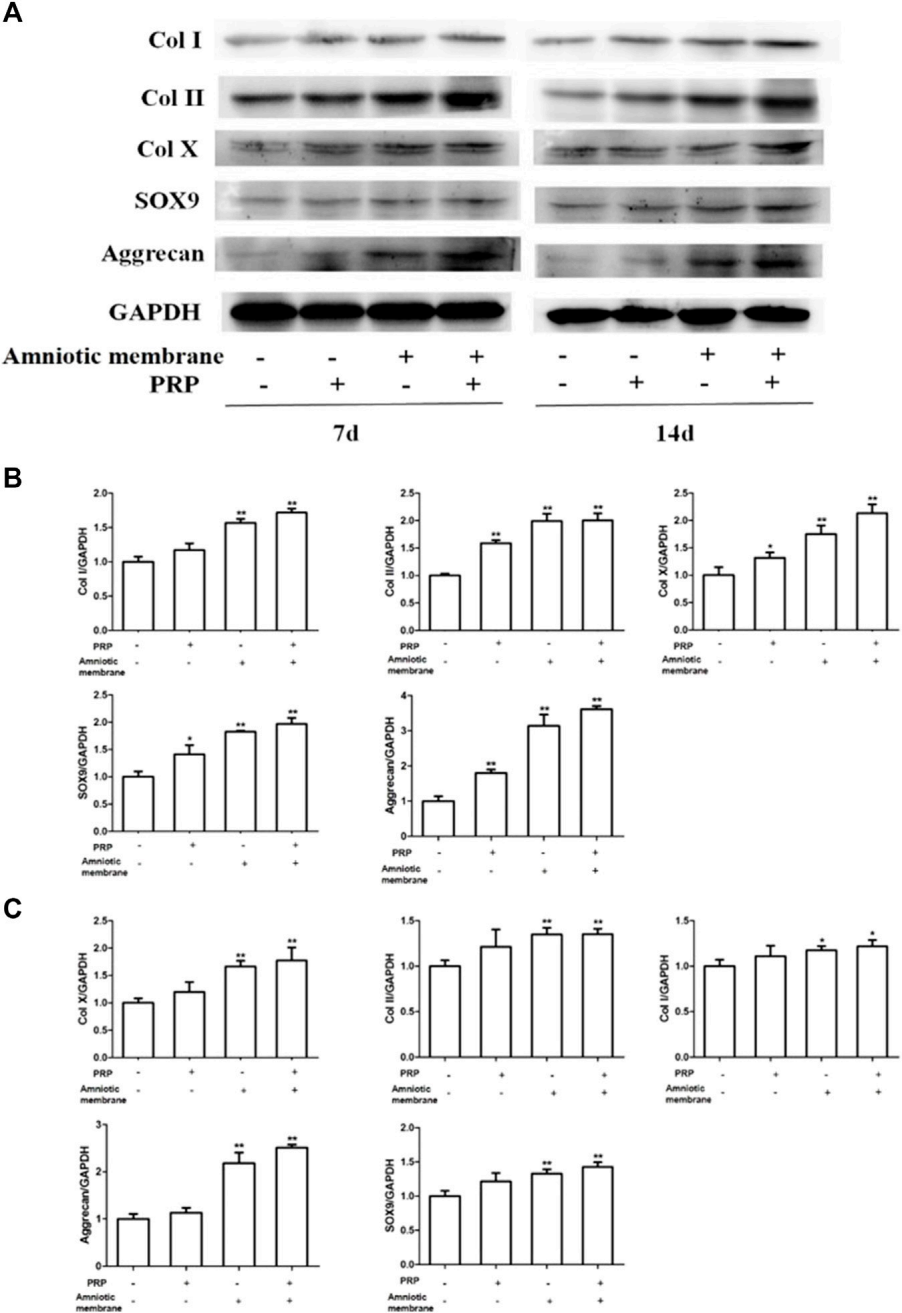
The combination of amniotic membrane and PRP scaffolds exhibited the most significant increase in the translational level of factors related to cell growth and differentiation in ADSCs. ADSCs were cultured under the treatment of different scaffolds. The protein expression levels of collagen I/II/x, Sox-9, and aggrecan were detected by Western blot on day 7 and day 14. **(A)** The detection of protein level of collagen I/II/x, Sox-9, and aggrecan by Western blot on day 7 and day 14. The expression of GAPDH was considered as the loading control. Two biological repeats were conducted in this part, and the most representative bands were shown for every targeted band. **(B,C)** The bar chart in this figure shows the quantification of the bands level of each gene on **(B)** day 7 and **(C)** day 14 in **(A)** by Lab image software. **p < 0.01, *p < 0.05.

The protein levels of *COL1A1*, *COL2A1*, *COL10A1*, *SOX9*, and *ACAN* in the four groups on days 7 and 14 were examined using Western blotting (Figures 4A–C). On days 7 and 14, the protein levels of *COL1A1, COL2A1, COL10A1, SOX9,* and *ACAN* in the ADSC + amniotic membrane + PRP group were significantly upregulated when compared with those in other groups (Figures 4A,B). Additionally, the protein expression levels of *COL1A1*, *COL2A1*, *COL10A1*, *SOX9*, and *ACAN* were comparable between days 7 and 14 (Figures 4A–C).

### Immunofluorescence Analysis of the Expression of COL1A1, COL2A1, COL10A1, SOX9, and ACAN

**FIGURE 5:**
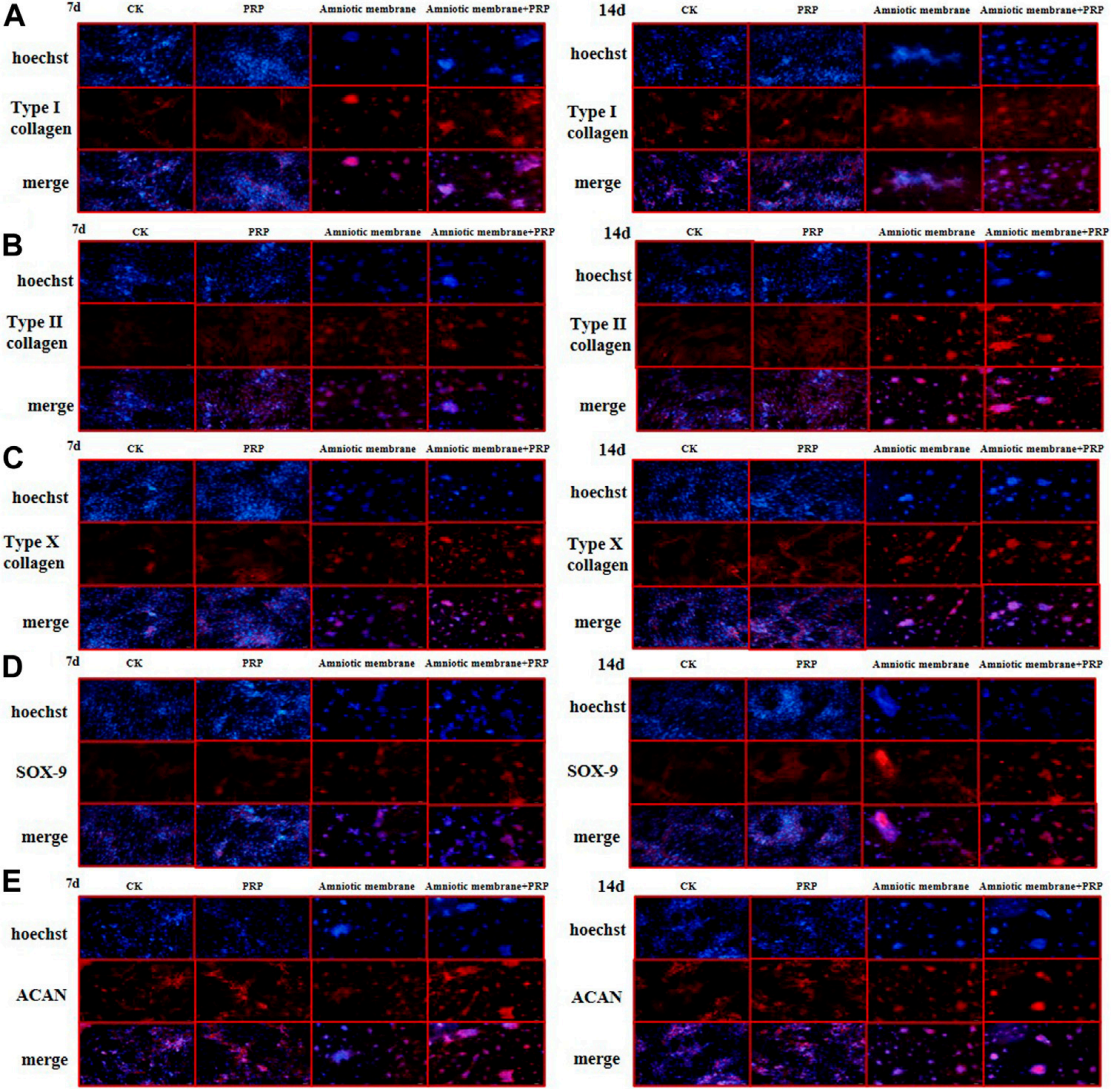
The protein expression levels of collagen I/II/X, Sox-9, and ACAN genes were the highest in ADSCs treated with amniotic membrane plus PRP scaffolds. The *in vitro* culture experiment of ADSCs was divided into four groups, including CK: ADSC alone; PRP: PRP + ADSC; amniotic membrane: amniotic membrane + ADSC; PRP + amniotic membrane: ADSC + PRP + amniotic membrane. Immunofluorescence tests were performed after 7 and 14 days co-culture. **(A–E)** The expression level of type **(A)** I collagen, **(B)** type II collagen, **(C)** type X collagen. **(D)** SOX-9, and, **(E)** ACAN in ADSCs cultured with amniotic membrane + PRP scaffolds on day 7 and day 14 was significantly higher than that in other groups, and the expression level on day 14 was significantly higher than that on day 7. The protein expression of each gene is amniotic membrane + PRP scaffolds group > amniotic membrane scaffolds group > PRP scaffolds group > CK group, *n* = 3.

The expression levels of *COL1A1*, *COL2A1*, *COL10A1*, *SOX9*, and *ACAN* in the ADSCs belonging to four experimental groups on days 7 and 14 were examined using immunofluorescence analysis. The ADSC + amniotic membrane + PRP group exhibited the highest expression levels of *COL1A1*, *COL2A1*, *COL10A1*, *SOX9*, and *ACAN*, followed by the ADSC + amniotic membrane, ADSC + PRP, and control groups (Figures 5A–E). In the ADSC amniotic membrane + PRP group, the expression levels of *COL1A1*, *COL2A1*, and *COL10A1* were markedly upregulated, which indicated that the combination of amniotic membrane and PRP promoted the differentiation of adipose stem cells into cartilage. The expression levels of *COL2A1* were higher than those of *COL1A1* and *COL10A1*, which indicated that collagen II accounted for a large proportion of cartilage.

### Wakitani Score

The Wakitani scores of the ADSC + PRP group (4.4 ± 0.44**) and ADSC + amniotic membrane group (2.63 ± 0.38**) were lower than those of the control group (6.733 ± 0.21) at week 12 post-operation. However, the Wakitani scores of the ADSC + amniotic membrane + PRP groups (1.33 ± 0.32**) were significantly lower than those of other groups (see Table 1 for further details) (* *p* < 0.05, ** *p* < 0.01, compared with the control group).

**TABLE 1:** Wakitani score: gross score of articular cartilage at 12 weeks after operation.

Group	General score
ADSCs group	6.733 ± 0.21
ADSCs + amniotic membrane group	2.63 ± 0.38**
ADSCs + PRP group	4.4 ± 0.44**
ADSCs + amniotic membrane + PRP group	1.33 ± 0.32**

The results showed that the scores of PRP group and amniotic membrane group were lower than those of ADSCs group at 12 weeks after operation, and the scores were further reduced after combined action of amnion and PRP. (*p < 0.05, **p < 0.01, compared with ADSCs group).

### Amniotic Membrane and PRP Combination Promoted Osteogenesis

**FIGURE 6:**
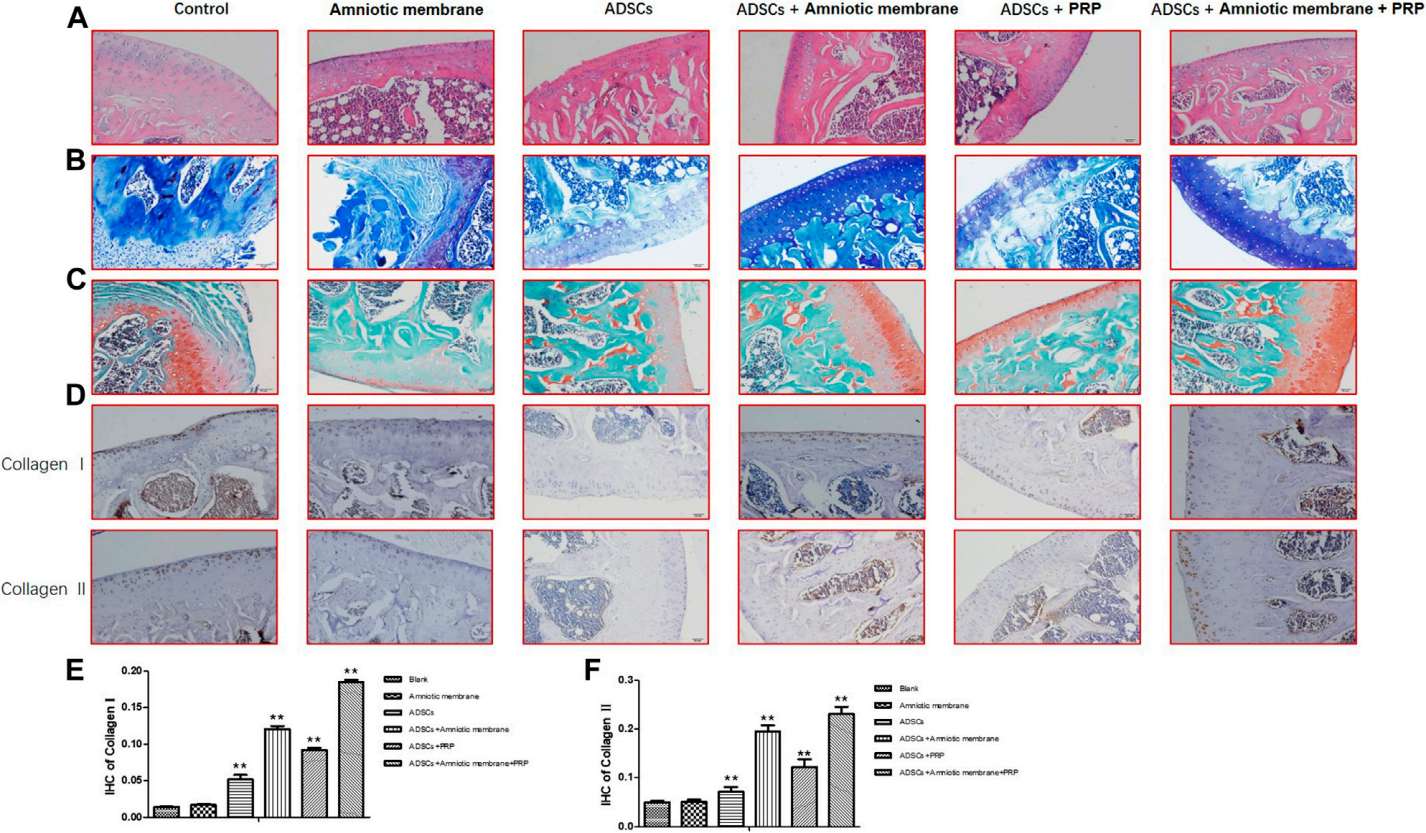
The combination of amniotic membrane and PRP scaffolds effectively induced the differentiation of ADSCs into cartilage. Three-month-old male New Zealand rabbits were used to establish an animal model of cartilage defect in the knee joint of rabbits. The model rabbits were divided into six groups, including control (PBS), ADSC, amniotic membrane, ADSC + amniotic membrane, ADSCs + PRP scaffolds, and ADSC + amniotic membrane scaffolds + PRP scaffolds were injected into the articular cartilage defect, respectively. Five rabbits were included in every group. After 12 weeks of normal culture, the repaired tissue were analyzed by biochemical staining for all six group of animals. **(A)** HE staining showed that the integrity of cartilage layer was higher in the PRP group, and the cartilage layer in amniotic membrane group and amniotic membrane + PRP group was basically restored; the bone formation area was enlarged. Compared with control and ADSC alone, the content of trabecular bone exhibited significant increase in amniotic membrane + ADSC, PRP + ADSC, and ADSC + PRP + amniotic membrane groups. *n* = 4. **(B)** Toluidine blue staining showed that membrane + ADSCs and ADSC + PRP + amniotic membrane groups showed deeper and more uniform staining. The surface of cartilage layer was smooth and the cells were arranged orderly. *n* = 3. **(C)** The results of saffron staining showed that the degree of cartilage damage in each group was in order from light to heavy: ADSC + amniotic membrane + PRP scaffolds group > amniotic membrane + ADSC > PRP + ADSC > ADSC > amniotic membrane and control group. **(D)** IHC staining results showed that the content of CollageI and CollageII proteins in the membrane + ADSC and ADSC + PRP + amniotic membrane groups were significantly increased compared with the other four groups, and the signals in the control group and amniotic membrane group were the least. Three sections were set for every treatment group, *n* = 6. **(E,F)** This diagram is the corresponding column diagram of CollageI **(E)** and CollageII **(F)** proteins in Figure D. (*p < 0.05, **p < 0.01, all groups were compared with the control group).

The HE staining results demonstrated that compared with those in the control group (PBS), the cartilage integrity and bone trabecular content were higher in the ADSC + PRP group. Compared with those in the ADSC + PRP group, the cartilage layer was markedly restored, the bone formation area was higher, the number of osteoclasts was significantly lower, and the bone trabecula content was higher in the ADSC + amniotic membrane and ADSC + amniotic membrane + PRP groups (Figure 6A). The number of chondrocyte in the ADSC + PRP group was significantly higher than that in the control group. Additionally, the chondrocytes exhibited orderly arrangement in the ADSC + PRP group. The cartilage layer staining intensity in the ADSC + amniotic membrane and ADSC + amniotic membrane + PRP groups was significantly higher than that in the ADSC + PRP group. The ADSC + amniotic membrane and ADSC + amniotic membrane + PRP groups exhibited smooth surface of cartilage layer and ordered arrangement of cells (Figure 6B). Safranin staining revealed that the control group exhibited the highest cartilage damage, followed by the ADSC + PRP, ADSC + amniotic membrane, and ADSC + amniotic membrane groups (Figure 6C). IHC analysis revealed that the levels of COL1A1 and COL2A1 in the ADSC + PRP group were significantly higher than those in the ADSC + PRP group. Additionally, the levels of COL1A1 and COL2A1 in the ADSC + amniotic membrane and ADSC + amniotic membrane + PRP groups were higher than those in the ADSC + PRP group (* *p* < 0.05, ** *p* < 0.01, compared with the control group) (Figures 6D–F).

### COL1A1, COL2A1, COL10A1, SOX9, and ACAN Were Upregulated in the Knee Joints of Rabbits Belonging to the ADSC + Amniotic Membrane + PRP Group

**FIGURE 7:**
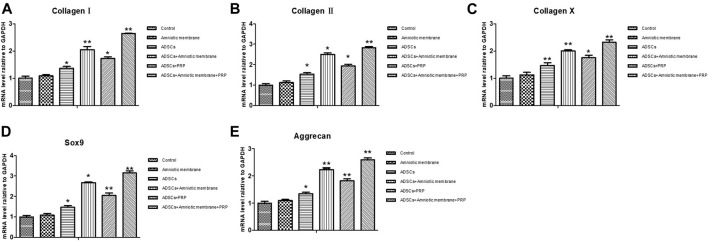
Amniotic membrane + PRP group showed the highest transcriptional levels of Col I, Col II, Col X, Aggrecan, and SOX9 genes in the extracellular matrix of cartilage. (**A–E)** RT-PCR was used to detect the mRNA expression of **(A)** collagen I, **(B)** collagen II, **(C)** collagen X, **(D)** Sox-9, and **(E)** aggrecan. The sequence of mRNA expression levels of these genes from high to low is as follows: ADSC + amniotic membrane + PRP group > ADSC + amniotic membrane group > PRP + ADSC group > ADSC > amniotic membrane and control group. Six groups were included for the analysis, including the control group, ADSC alone, amniotic membrane alone, ADSC + amniotic membrane, ADSCs + PRP scaffolds, and ADSCs + amniotic membrane + PRP. Five rabbits were included for every group and the extracellular matrix of cartilage was isolated, respectively.

**FIGURE 8:**
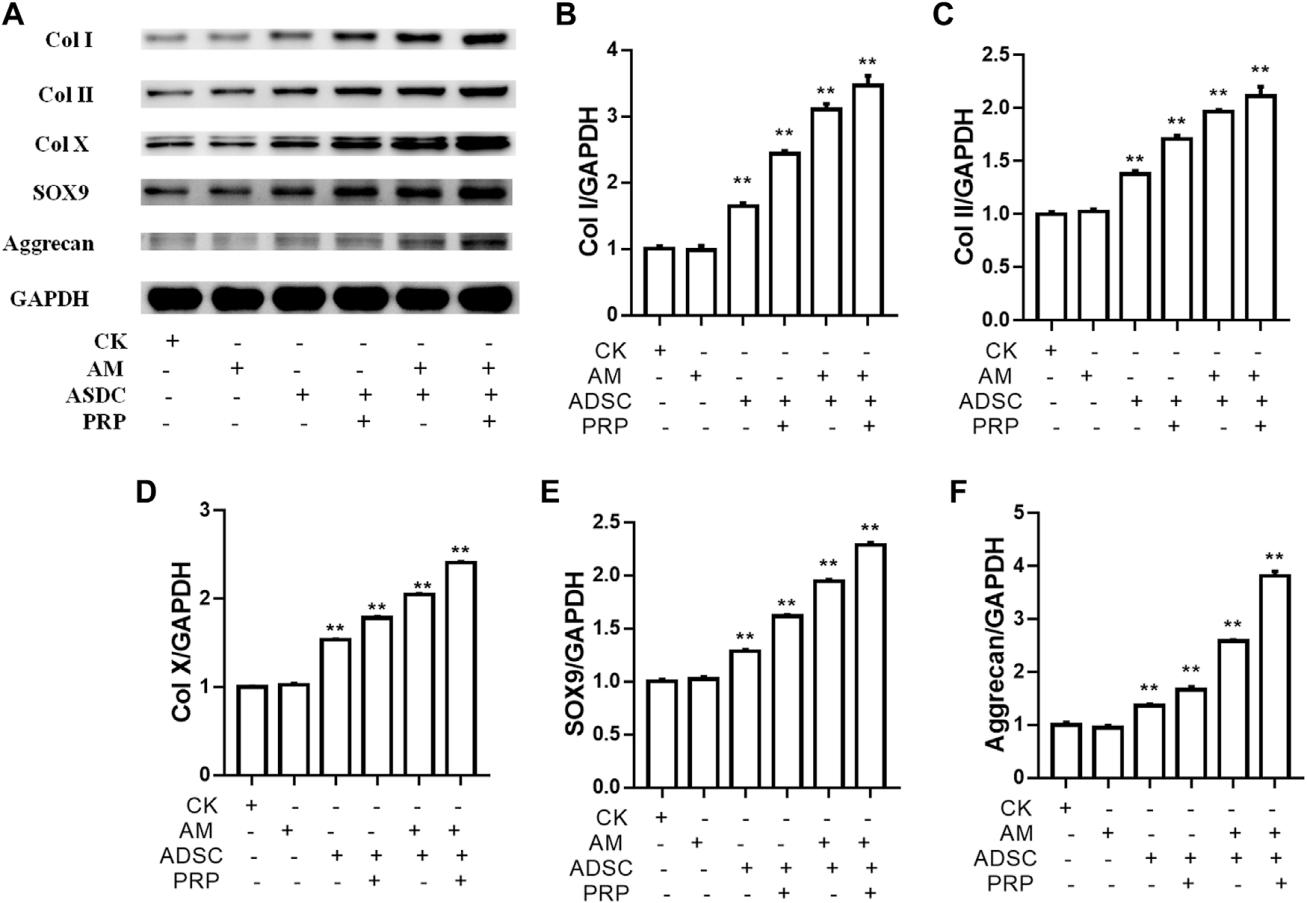
Amniotic membrane + PRP group showed the highest translational levels of Col I, Col II, Col X, Aggrecan, and SOX9 genes in the extracellular matrix of cartilage. **(A)** Western blot test was used to detect the expressions of collagen I/II/X, Sox-9, and Aggrecan. The sequence of protein expression levels of these genes from high to low is as follows: ADSC + amniotic membrane + PRP group > ADSC + amniotic membrane group > PRP + ADSC group > ADSC > amniotic membrane and control group (*p < 0.05, **p < 0.01; a *p*-value of less than 0.05 was considered as statistically significant). Six groups were included for the analysis, including the control group, ADSC alone, amniotic membrane alone, ADSC + amniotic membrane, ADSC + PRP, and ADSC + amniotic membrane + PRP. Five rabbits were included for every group and the extracellular matrix of cartilage was isolated for protein extraction. Ten micrograms of protein was loaded and GAPDH was considered as the loading control, **(B–F)** The quantification of **(B)** collagen I, **(C)** collagen II, **(D)** collagen X, **(E)** Sox9, and **(F)** aggrecan for the bands in **(A)** (*p < 0.05, **p < 0.01; a *p*-value of less than 0.05 was considered as statistically significant).

**FIGURE 9:**
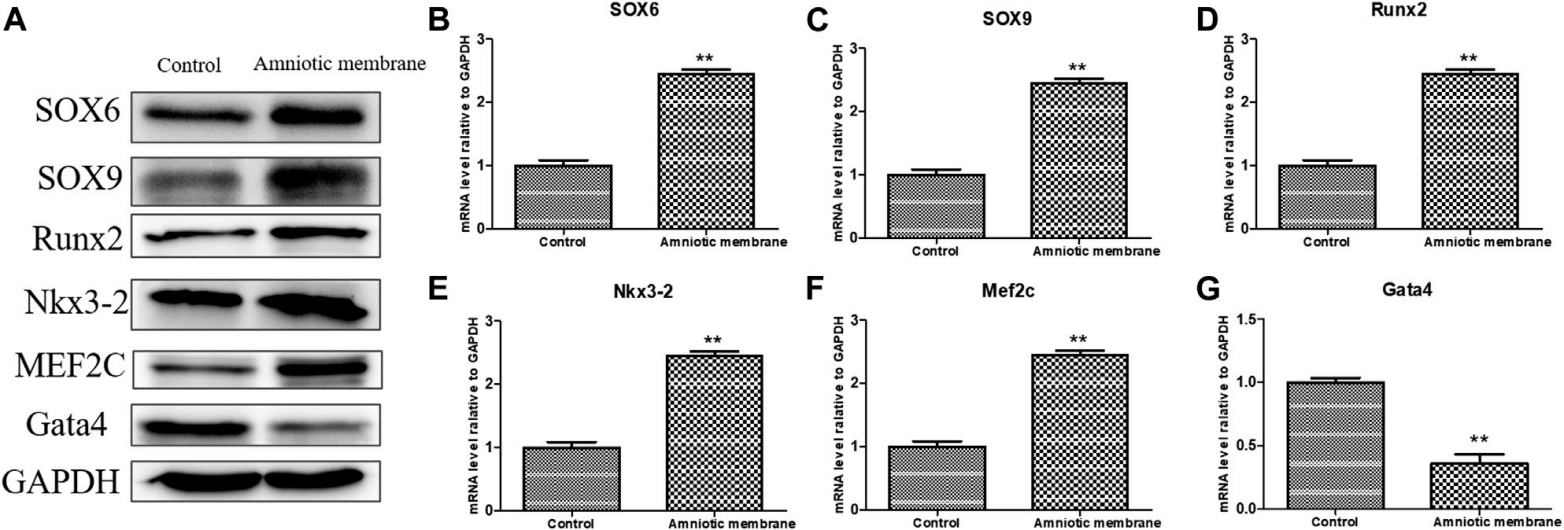
The effect of amniotic membrane on transcription and translation levels of Runx2, Aggrecan, Nkx3-2, MEF2C, Gata4, SOX6, and SOX9 genes. **(A)** The expression levels of Runx2, Aggrecan, Nkx3-2, MEF2C, Gata4, SOX6, and SOX9 under amniotic membrane treatment were detected by Western blot. The expression of GAPDH was considered as the loading control. **(B–G)** The mRNA level of **(B)** SOX6, **(C)** SOX9, **(D)**Runx2, **(E)** Nkx3-2, **(F)** MEF2C, and, **(G)** Gata4 in the control and amniotic membrane treatment group, *n* = 3. **p < 0.01, *p < 0.05.

The knee joints of New Zealand were subjected to qRT-PCR and Western blotting analyses at week 12 post-operation. The ADSC + PRP + amniotic membrane group exhibited the highest mRNA levels of *COL1A1*, *COL2A1*, *COL10A1*, *SOX9*, and *ACAN*, followed by the ADSC + amniotic membrane, ADSC + PRP, and control groups (** *p* < 0.01; Figures 7A–E). Consistently, Western blotting analysis revealed that the ADSC + PRP + amniotic membrane group exhibited the highest protein levels of COL1A1, COL2A1, COL10A1, SOX9, and ACAN, followed by the ADSC + amniotic membrane, ADSC + PRP, and control group (* *p* < 0.05 and ** *p* < 0.01; Figures 8A–F). Next, the effect of amniotic membrane on the level of cartilage regeneration-related factors (SOX6, SOX9, RUNX2, NKX3-2, MEF2C, and GATA4) was examined. The expression levels of SOX6, SOX9, RUNX2, NKX3-2, MEF2C, and GATA4 were upregulated in the amniotic membrane group (Figures 9A–G).

### Scaffolds Comprising the Combination of Amniotic Membrane and PRP Affected the Secretion of Cytokines in the ADSCs *In Vivo*

**FIGURE 10:**
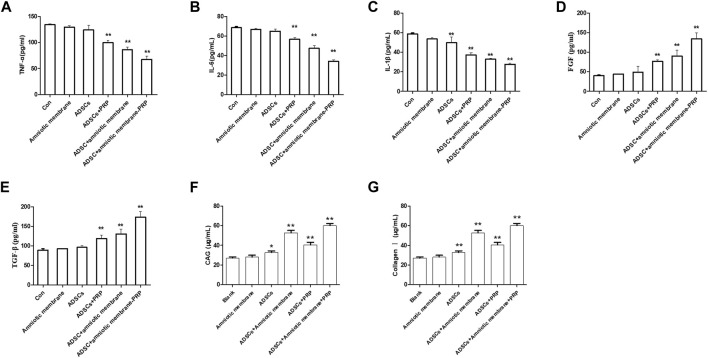
The combination of amniotic membrane and PRP scaffolds upregulated the levels of glycosaminoglycan, collagen, TGF-β, PDGF, and FGF while decreased the level of IL-1β, IL-6, and TNF-α. **(A–C)** The detection of the levels of **(A)** TNF-α, **(B)** IL-1β, and **(C)** IL-6 in different groups by ELISA assay. Six groups were included for the analysis, including the control group, ADS alone, amniotic membrane alone, ADSC + amniotic membrane, ADSC + PRP, and ADSC + amniotic membrane + PRP. Five rabbits were included for every group (*p < 0.05, **p < 0.01, all groups were compared with the control group; a *p*-value of less than 0.05 was considered as statistically significant). **(D–G)** The detection of **(D)** FGF, **(E)** TGF-β, **(F)** glycosaminoglycan, and **(G)** collagen Ⅰ in different groups by ELISA assay. Six groups were included for the analysis, including the control group, ADSC alone, amniotic membrane alone, ADSC + amniotic membrane, ADSC + PRP, and ADSC + amniotic membrane + PRP. Five rabbits were included for every group (*p < 0.05, **p < 0.01, all groups were compared with the control group; a *p*-value of less than 0.05 was considered as statistically significant).

The articular cartilage and articular cavity of New Zealand rabbits were subjected to ELISA at week 12 post-operation. The control group exhibited the highest levels of IL1β, IL6, and TNFα, followed by the ADSC + PRP, ADSC + amniotic membrane, and ADSC + PRP + amniotic membrane groups (* *p* < 0.05, ** *p* < 0.01; Figures 10A–C). The ADSC + PRP + amniotic membrane group exhibited the highest articular cavity levels of FGF, TGFβ, GAG, and COL1A1, followed by the ADSC + amniotic membrane, ADSC + PRP, and control group (* *p* < 0.05, ** *p* < 0.01; Figures 10A–C).

## Discussion

The amniotic membrane is a translucent membrane with a smooth inner layer and without blood vessels, nerves, or lymph. The amnion has wide applications as a biomaterial in basic research and clinical studies owing to its unique structural and functional properties (Lacorzana, 2020). The amniotic membrane contains type I, II, and III collagen and elastin, which can resist certain tensions and exhibit elastic properties (Fenelon et al., 2019). As amniotic membranes exhibit anti-inflammatory, antibacterial, antiviral, and low immunogenic activities, they are ideal grafts (Farhadihosseinabadi et al., 2018). The amniotic membrane can physically prevent the infiltration of inflammatory cells, attract and trap inflammatory cells, and consequently inhibit the release of inflammatory mediators (Duerr et al., 2019). Amniotic membrane transplantation can prevent the formation of dead space and reduce the accumulation of secretions. Collagen fibers in the matrix exert hemostatic effects, which can prevent the formation of postoperative hematoma and reduce the risk of infection (Jirsova and Jones, 2017). Some antimicrobial peptides, such as defensin, elastin, and secretory leukocyte protease inhibitors, which exert anti-inflammatory and antimicrobial effects, are expressed in the amniotic membrane. Amniotic membranes promote epithelial formation and inhibit fibrosis. Additionally, amniotic membrane is a good extracellular matrix as it can enhance the adhesion of epithelial cells, promote cell proliferation and migration, inhibit apoptosis, and promote epithelial formation (Niknejad et al., 2016). Bioactive components, such as epidermal growth factor (EGF), keratinocyte growth factor (KGF), hepatocyte growth factor, and fibroblast growth factor (bFGF) in the amnion promote wound healing. In particular, EGF and KGF, which can promote the proliferation and migration of epithelial cells, play an important role in wound healing (Stachon et al., 2017; Pogozhykh et al., 2020).

In this study, the combination of amniotic membrane and PRP exerted the highest chondrogenic effect. However, the chondrogenic effect of amniotic membrane was higher than that of PRP. PRP scaffolds can release various factors that promote the proliferation and differentiation of stem cells during stem cell culture. The levels of cytokines peaked on day 14 and decreased thereafter (Figure 2B). This indicated that the PRP scaffolds could not promote the growth and differentiation of stem cells. Electron microscopy analysis revealed that the compactness and concave and convex space areas of the amniotic membrane scaffolds were higher than those of the collagen scaffolds (Figure 2A). This enables the attachment and growth of stem cells. Additionally, the concave space protects the cells. *In vitro* culture of ADSCs from New Zealand rabbits revealed that the chondrogenesis of the amniotic membrane and PRP scaffolds was higher than that of the amniotic membrane or PRP scaffolds. This may be because the amnion provides an effective attachment point for stem cells and releases various factors than promote proliferation and differentiation. The knee joint injury repair in New Zealand rabbits involves the proliferation and differentiation of ADSCs *in vivo*. The Wakitani score was the lowest in the ADSC + amniotic membrane + PRP group, which indicated enhanced knee joint repair efficiency (Table 1). HE staining revealed that the injured cartilage layer significantly recovered in the ADSC + amniotic membrane and ADSC + amniotic membrane + PRP groups, which exhibited decreased number of osteoclasts and increased trabecular bone content (Figure 6A). These findings indicate that the amniotic membrane scaffolds can promote the formation of cartilage and bone. During the 12-month feeding of New Zealand rabbits, the amniotic membrane scaffolds may release many factors and prolong their activity. Toluidine blue staining revealed that the ASDC + amniotic membrane and amnion + PRP groups exhibited the highest staining intensity. Additionally, the surface of the cartilage layer was smooth and the cells exhibited ordered arrangement in the ASDC + amniotic membrane and ASDC + amniotic membrane + PRP groups (Figure 6B). Safranine O staining revealed that the ASDC + amniotic membrane and amniotic membrane + PRP groups exhibited the lowest degree of cartilage damage. These results indicated that the amniotic membrane promoted the proliferation and differentiation of ADSCs and that it could effectively alleviate joint injury in animals (Figure 6C).

In this study, the expression levels of COL1A1, COL2A1, COL10A1, SOX9, ACAN, TGFβ, PDGF, and FGF in ADSCs and their extracellular matrix after chondrogenesis were the highest in the ASDC + amniotic membrane + PRP groups *in vitro* and *in vivo*, followed by the ADSC + amniotic membrane, ADSC + PRP, and control groups. Type I collagen, which is expressed in the bone and skin (Figures 3–5, 7, 8B), accounts for more than 90% of bone organic matter. Additionally, type I collagen is the main component of the skin, tendons, and ligaments. Type II collagen is the main cartilage collagen (Tiku and Madhan, 2016). Pure type II collagen forms a complex with glucosamine and chondroitin (Nagaoka et al., 2019). Type X collagen, a type of short-chain non-fibrous collagen, comprises three identical α1 chains and is expressed in the matrix around the chondrocytes in the cartilage center and epiphyseal cartilage growth plate (Knuth et al., 2019). The enhanced levels of these collagen types promote the repair of the damaged joints. SOX9, an important factor in chondrocyte differentiation, is an early marker of chondrocyte differentiation. Previous studies have reported that SOX9 can induce the expression of type II collagen and GAG (Maneix et al., 2014). Fujita et al. (2010) demonstrated that the expression levels of SOX9, type II collagen, and type X collagen was upregulated in the side of ankle process resection at week 4 post-ankle process resection. The expression of SOX9 and different collagen types was hypothesized to promote cartilage regeneration. The core protein of aggrecan, a proteoglycan originally isolated from cartilage tissue, comprises several domains and approximately 100 chondroitin sulfate chains (Morawski et al., 2012). Cosenza et al. (2017) isolated mouse bone marrow mesenchymal stem cells using differential centrifugation to examine the role of MPS and exosomes in osteoarthritis. MPS and Exos promoted the expression of type II collagen and aggrecan in chondrocytes and effectively repaired the joint injury in mice.

TGF-β, which belongs to the TGF-β superfamily, regulates cell growth and differentiation. The level of TGF-β is upregulated in tissues undergoing active cell differentiation. PDGF, a basic protein stored in platelet α granules, can stimulate cells in the G0/G1 phase to enter the division and proliferation cycle (Cucchiarini et al., 2018). FGF can promote the migration of endothelial cells and the proliferation of smooth muscle cells, but cannot promote the migration of smooth muscle cells. Additionally, FGF can promote the formation of the cardiovascular system and repair the damaged endothelial cells. In the skeletal system, FGF can promote the generation of osteoblasts (Adhikary et al., 2019). IL-1β, a cytokine of the chemokine family, is involved in the pathogenesis of osteoarthritis. Previous studies have reported that IL-1β can stimulate chondrocytes and synovial cells to produce proteases, such as matrix metalloproteinases (MMPs), downregulate the expression of MMP-3 inhibitor, promote the degradation of cartilage matrix, stimulate chondrocytes and synovium to produce nitric oxide (NO), inhibit the proliferation of chondrocytes, and induce apoptosis of chondrocytes (Ma et al., 2019). IL-6, a lymphokine produced by activated T cells and fibroblasts inhibits osteogenic differentiation and fracture healing TNF-α, which is mainly produced by activated monocyte macrophages and other types of cells, can inhibit the osteogenic differentiation of stem cells (Johnson et al., 2017).

In this study, ADSCs were analyzed *in vitro* and *in vivo*. The scaffolds comprising the combination of amniotic membrane and collagen scaffolds promoted the proliferation and differentiation of ADSCs and effectively repaired the damaged joints. Amniotic membrane played an important role in the entire repair process. The combination of amniotic membrane and collagen scaffolds provides an effective medium for stem cell growth for articular cartilage cell transplantation and regeneration. Thus, this combination is an effective biomaterial for researchers and clinicians.

## Data Availability

The original contributions presented in the study are included in the article/Supplementary Material, further inquiries can be directed to the corresponding authors.
