# Supplementary material for: Effect of Amniotic Membrane/Collagen-Based Scaffolds on the Chondrogenic Differentiation of Adipose-Derived Stem Cells and Cartilage Repair
Source: Front Cell Dev Biol. 2021 Nov 25;9:647166. doi: 10.3389/fcell.2021.647166 (PMC8657407; doi:10.3389/fcell.2021.647166)
Supplement: Supplementary file 1 [file Image1.pdf]

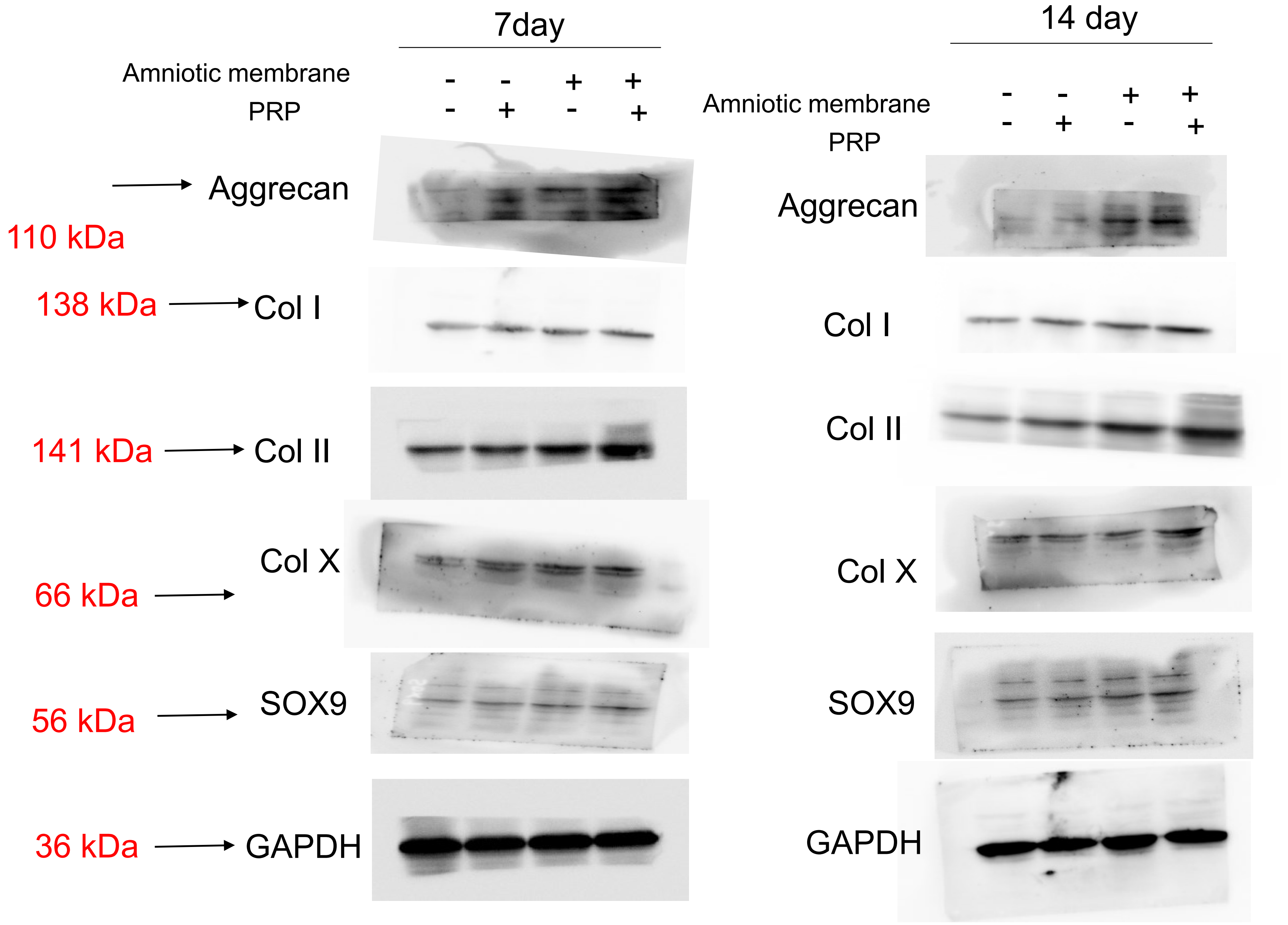

Figure S1. The original bands for figure 4A

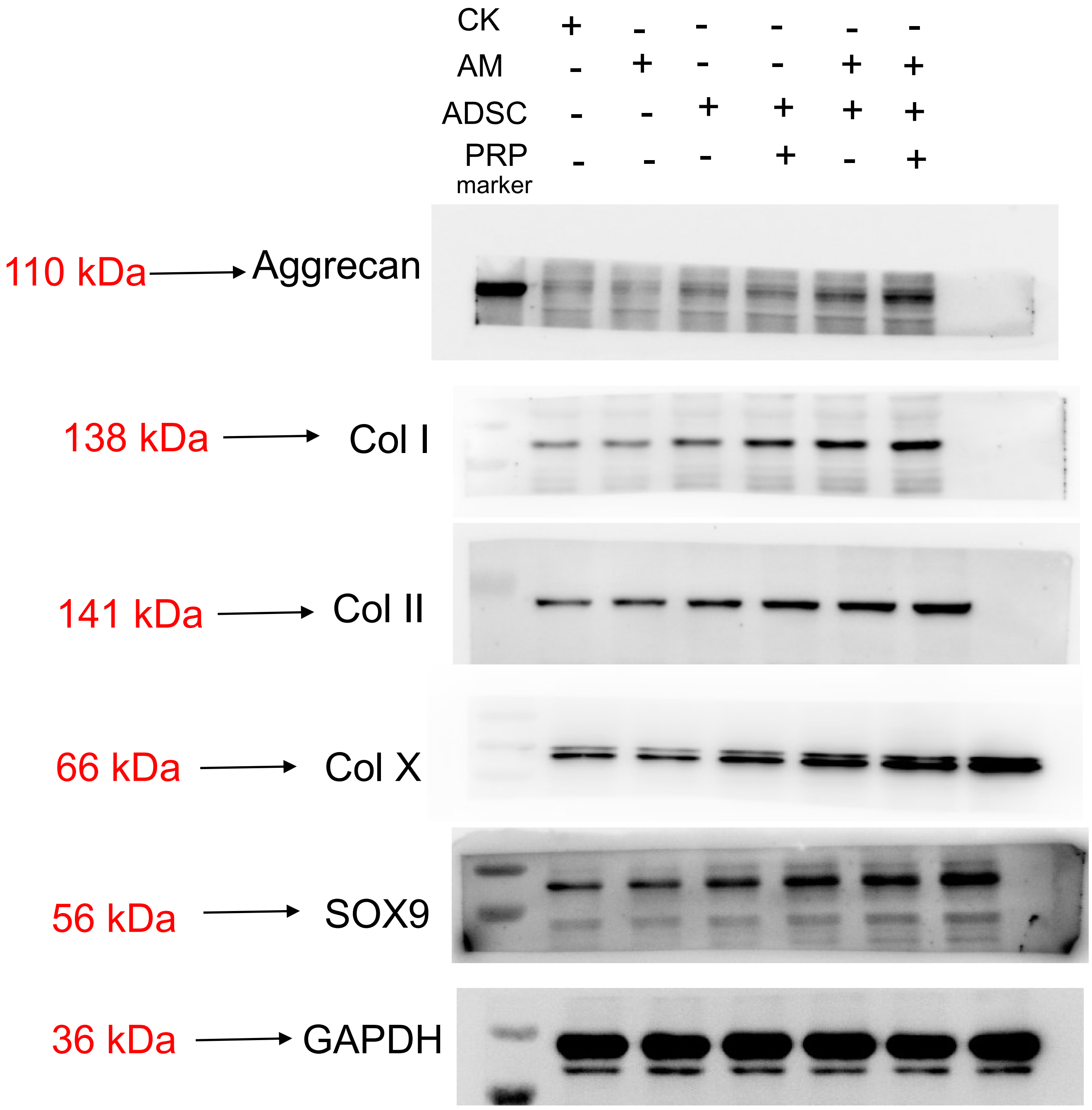

Figure S1. The original bands for figure 8A

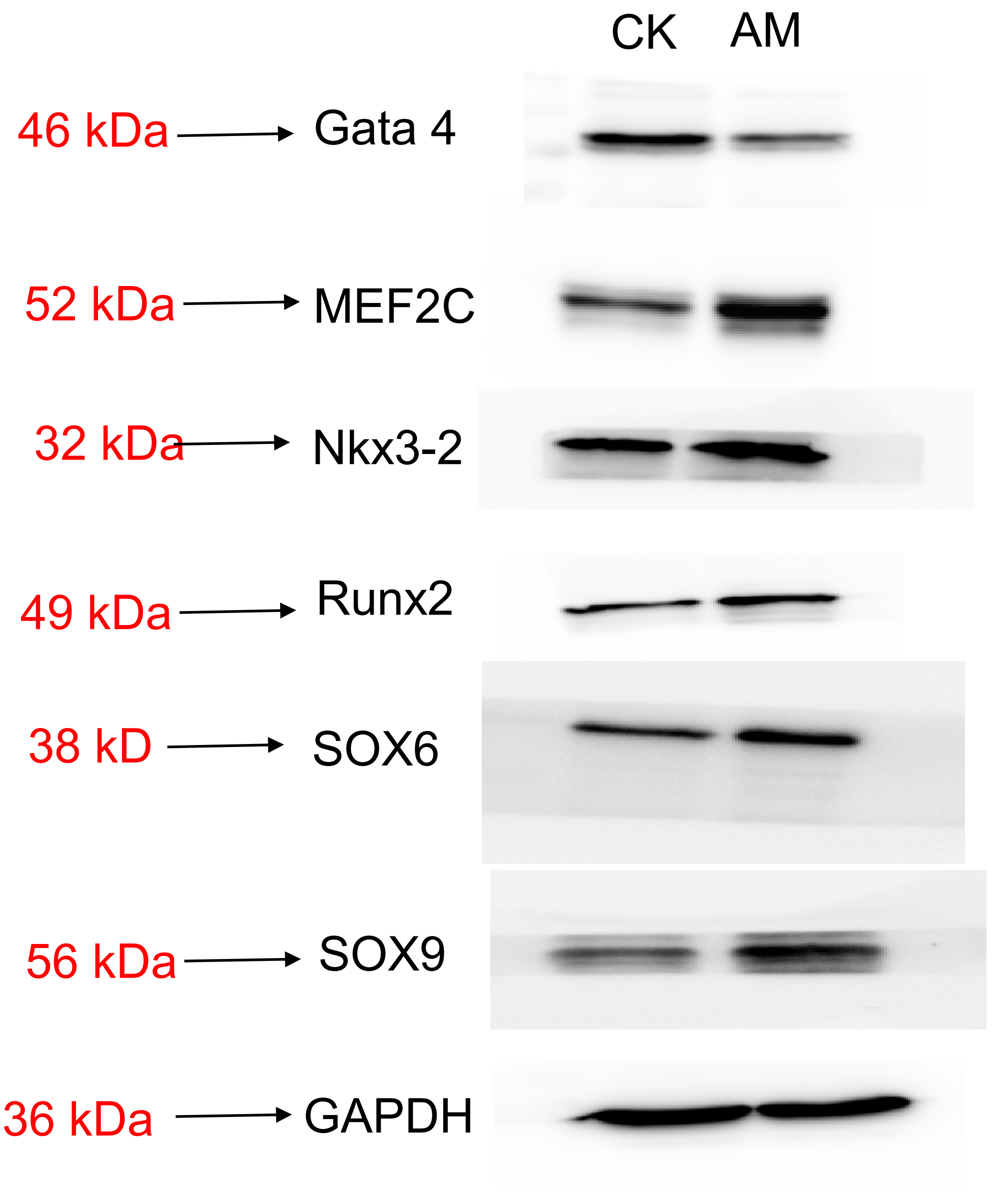

Figure S3. The original bands for figure 9A
